# An analysis of patient house calls in the area of Attica, Greece

**DOI:** 10.1186/1472-6963-6-112

**Published:** 2006-09-05

**Authors:** George Peppas, George Theocharis, Efthymia A Karveli, Matthew E Falagas

**Affiliations:** 1 Alfa Institute of Biomedical Sciences (AIBS), Athens, Greece; 2 SOS doctors, Athens, Greece; 3 Department of Medicine, Tufts University School of Medicine, Boston, Massachusetts, USA

## Abstract

**Background:**

Physicians house calls to patients have been declining in many countries of the world. The objective of this study was to describe the patterns of utilization of house call services provided by SOS doctors in the area of Attica, Greece.

**Methods:**

We performed a retrospective analysis of data regarding house call visits of patients in the area of Attica (metropolitan area of Athens and surrounding cities), Greece. Characteristics of patients who received house call services by a physician, including diagnosis, and recommended management plan, including advice for hospitalization were analyzed.

**Results:**

SOS doctors in the area of Attica, Greece performed 98,009 house calls during the 5-year study period (1/11/2000 – 31/10/2005). Patients older than 65 yeas requested 47.8% of the house calls. Females requested more house calls during the studied period compared to males (59.4% versus 40.6%, p < 0.001). The majority of the diagnoses (18.4%) were infections of the upper and lower respiratory tract. 9.1% of patients were advised after the evaluation at home to be admitted to a hospital to receive inpatient services.

**Conclusion:**

Our analysis documents the utilization of house calls by SOS doctors in the area of Attica, Greece during a 5-year period. We believe that house calls provided by individual practitioners, physician group practices, or organized companies and organizations should re-gain their well-deserved position in the modern health care systems.

## Background

Physicians' patient house calls have been traditionally considered a vital part of an integrated health care system [1,2]. However, this type of medical care service has been declining for decades in several parts of the world, as shown by several studies that have been performed in various countries, such as the USA, Canada, and Slovenia [3-6]. It is interesting that in the USA, about 40% of physician-patient encounters involved house calls during the 1930s in contrast to less than 1% between 1998–2004 [7,8].

There are several reasons that may explain the decreasing utilization of medical house call services. These may include improvement in medical transportation by ambulances, upgraded and efficient hospital facilities and their emergency departments, increased cost of house calls, the doctors' fear of personal safety, and the doctors' busy and/or complicated work schedule [2,8]. Also, patients may think that medical records are appropriately kept in hospitals and thus, hospital doctors may find the records readily available for use, if needed. It is possible that the availability of information regarding the history and previous medical interventions and their result makes the treatment of patients with chronic disease more efficient. In addition, when seeing a patient within the hospital environment, the physicians have more opportunities of asking a colleague for a second opinion or even consult another specialist on a doubtful diagnosis. Finally, when making house calls, the physician has to practice medicine without having readily available useful diagnostic recourses and therapeutic interventions, facts that may be proven to be of significance in a complicated medical case [9]. This is another reason that may make patients to feel safer receiving care in a hospital environment.

On the other hand, patients seem to appreciate the physicians' house calls because sometimes their condition makes it difficult to move, especially if the medical problem arises at night hours [7,10]. In addition, patients frequently seek for a more personal approach by the doctor, something that may be more easily accomplished in the home than the hospital environment [9].

The decline of physicians' visits at patients' homes has generated the need for organized home care by various medical organizations and companies in several countries [11]. SOS doctors, Athens, Greece is a medical company that provides fee for service, emergency, outpatient services including house calls, in the area of Attica, Greece. In this paper, we analyzed the reasons for the physicians' medical house calls, the characteristics of the patients, as well as the services provided.

There are some characteristics of SOS doctors in Greece that need to be mentioned in order to better understand the provided medical services. First, house calls are made not only by general practitioners but also by specialized doctors. This becomes possible because of the availability of such doctors due to the large number of physicians per capita in Greece. The services of 21 different medical specialties are available in the Greek organization of SOS doctors. Second, an effort is being made to develop a relationship between the physician and the patient by trying to send the same doctor to a given patient after the first house call, yet the SOS doctors do not see patients only in the context of the organization, as nearly everyone has his/her private patients who are not included in the number of SOS doctors visits. Third, patient medical records of the visits are meticulously kept and are available to doctors making the house calls. Fourth, about 70% of house calls are performed within one hour from the request. Fifth, doctors making house calls are equipped with basic drugs as well a machine for electrocardiograms (ECCs).

## Methods

### Patient population

The study population was comprised of patients who requested medical services by the SOS doctors in the area of Attica, Greece, that is inhabited by 3,894,573 people (population census March 18, 2001), during the period 1/11/2000 – 31/10/2005, which, at the time of the study, was the most recent one of available data. A physician is available 24 hours a day, 365 days a year, to answer phone calls of patients who request medical services. He or she makes the initial assessment whether the patient should be seen by one of SOS doctors (and what specialty) or should request ambulance for immediate transfer to a hospital. If a patient is seen by a SOS doctor, a specially designed form is filled by this doctor including data regarding the chief complaint, history of present illness, past medical and surgical history, allergies, findings of the physical examination, assessment based on the history and examination, likely diagnosis, and management plan (that may include recommendation for admission to a hospital, laboratory and/or imaging tests, medical and/or surgical treatment, and re-evaluation). The study was approved by the Ethics Committee of SOS doctors, Greece. No individual informed consent was obtained

### Data analysis

The available data regarding home visits by SOS doctors during the study period were stratified based on demographic characteristics of the patients (age and sex) and time of the visit (time within the day, month, season, and year) to analyze patterns of utilization of this health care service. In addition, the data were analyzed to evaluate the diagnosis (in groups of diseases) as well as the recommendations provided to the patients by the SOS doctors especially regarding the need for hospitalization.

### Statistical methods

The distribution of variables in the compared groups was done with X^2 ^or Fischer exact test for categorical variables and Student's t-test or the Mann – Whitney test for normally and non-normally distributed continuous variables, respectively. Statistical analyses were performed using a version 13.0 SPSS software program (SPSS Inc., Chicago, Illinois, USA).

## Results

**Figure 1:**
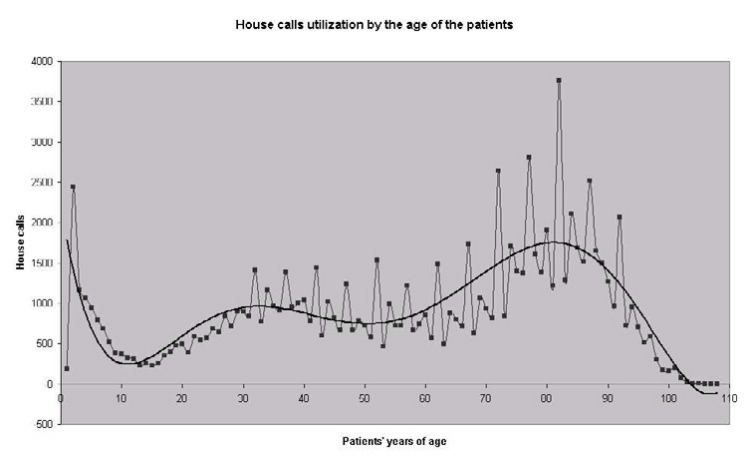
House calls utilization by the age of the patients.

**Figure 2:**
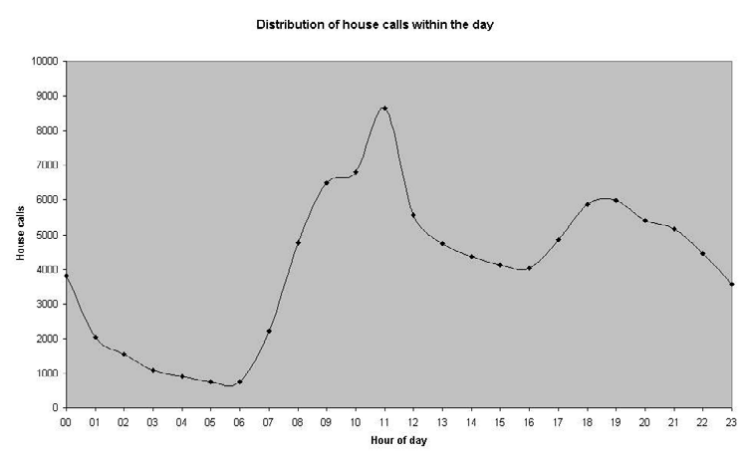
Distribution of house calls within the day.

During the 5-year study period SOS doctors performed 98,009 house calls in the area of Attica, Greece. In Table 1 we present data regarding the age, and sex of the patients as well as the distribution of the visits in different time periods. Patients older than 65 years requested 47.8% of the house calls. Females requested more house calls during the studied period compared to males (59.4% versus 40.6%, p < 0.001). In Figure 1 we present the utilization of SOS doctors home medical service stratified by the age of the patients, where it is clearly shown that the highest utilization of this emergency medical service was performed by patients aged between 75 and 90 years, while the group of children aged less than 3 years follows (data were not adjusted for population). In Figure 2 the distribution of house calls within the day is shown. The highest number of house calls was done about 11 am, while at 6:30 pm the curve presents the second highest peak, which demonstrates the high request of SOS-doctors emergency medical service at that hour as well. The house calls performed between 4 pm and midnight are 86% of the morning shift (8 am – 4 pm) house calls.

**Figure 3:**
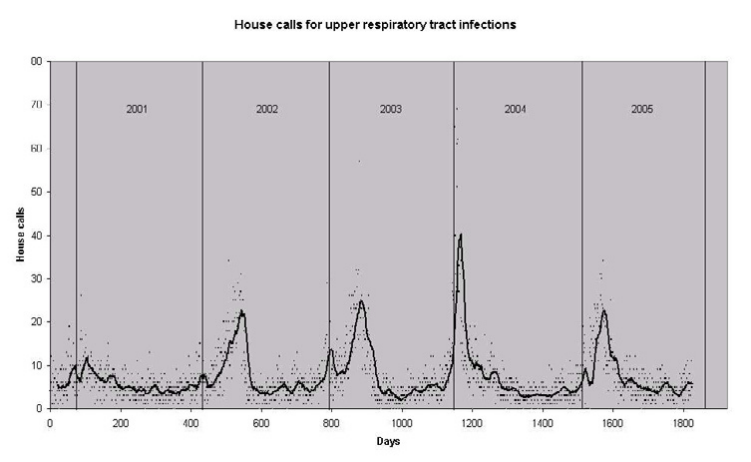
House calls for upper respiratory tract infections.

In Table 2 we present data regarding the working diagnosis after the evaluation of the patients based on information from the history and findings from the physical examination. The majority of the diagnoses (18.4%) were infections of the upper and lower respiratory tract. 9.1% of patients were advised after the evaluation at home to be admitted to a hospital to receive inpatient services. Cardiovascular disorders were the second most likely cause (9.4%) of requests for house calls during the study period. Psychiatric disorders were the reason for 3.6% of the requests for house calls, placing them in the sixth place on the relevant list. Finally, in Figure 3 we present the distribution of upper respiratory tract infections in the 5-year period examined. Peaks of the curve are noted in the first trimester of each year, representing the outbursts of upper respiratory tract infections occurring during this time.

## Discussion

In this paper we report the patterns of utilization of house calls by SOS doctors in the area of Attica, Greece, during the period 1/11/2000 – 31/10/2005. In accordance to other studies, our analysis revealed that the elderly and the female patients requested more house calls during the period examined compared to younger and male patients, respectively [12,13]. It is also worth noting that a considerable proportion of house calls were performed from 4 pm – 12 pm (40.1%) and midnight – 8 am (13.4%), a finding also observed in other relevant studies [14,15]. These data show that the SOS doctors' house call service accommodates patients with urgent health problems during periods of the day when the majority of physicians' offices are closed.

Our analysis shows that infections of the upper and the lower respiratory tract were the most likely cause of requesting house call services by the SOS doctors in Attica. Also, the secular trends of the distribution of house calls due to upper respiratory tract infections clearly depict the epidemic nature of these infections. We noted one or two peaks of requests for management of upper respiratory tract infections for each year during the 5-year study. It is interesting that these peaks occurred during different times in winter and early spring (from December to March). Public health authorities may find useful the real-time, ongoing collection of data regarding house calls by SOS doctors for the purpose of surveillance of infectious diseases.

The low utilization of SOS doctors' house calls during September deserves to be emphasized. We should acknowledge that there is no clear explanation for this observation. An effect of summer vacations (that are taken by most Greeks during August) on morbidity is an intriguing possibility. It is likely that the physical and psychological rejuvenation, change of environment, and increase of physical exercise during vacation may have a considerable effect on morbidity.

Another noteworthy finding of our analysis is that the services provided by SOS doctors in Attica, Greece have been extremely valuable during the vacations periods. This is especially true in August when a considerable proportion of hospital doctors take their annual leave at a time when the number of tourists is high in Greece, including Attica where the metropolitan area of Athens is located.

Although the practice of making medical house calls has diminished significantly during the last 30–40 years in many parts of the world, they remain an important component of the health care system in several countries. The most representative example is Great Britain where general practice, including patient house calls, is one of the factors that distinguish primary care in Britain, from that in many other western countries [16]. It seems that is in the British culture for patients to expect their family physician to make house calls and for doctors to agree that house calls are valuable for good patient care. It should be emphasized that the house calls that are declining throughout the world are mostly those done during regular hours by personal doctors. However emergency services that SOS doctors provide are another organizational form.

The major provider of health care home services in Greece is IKA (the major public heath insurance provider of the country) that offer the possibility of house calls by general practitioners within the same day and mainly during working hours. We do not know the number of patients examined daily via this services system.

Our study is not without limitations. It is a retrospective analysis with all limitations inherent to this study design. Thus, the research questions were formulated after the data had been collected in a routine fashion, a fact that is sometimes associated with missing information for some variables for a proportion of patients included in the analysis. We acknowledge that a study using a prospective design would offer the opportunity for additional analyses. Research questions regarding the occurrence of various diseases during different hours of the day or during different months and seasons of the year would offer interesting data of higher quality compared to those of a retrospective study. Such circadian and seasonal differences in the incidence of various diseases may have potential pathophysiological implications and clinical importance.

Although we tried to retrieve annual data, unfortunately there was no information regarding the population of the area of Attica for each of the years 2000–2005 separately. The Greek population census takes place every 10 years. The last population census took place in Greece on March 18, 2001, and, according to that data, Attica was inhabited by 3,894,573 citizens [17]. Therefore, it was not possible to further describe the trends in utilization over the 5-year period for specific subsets of population.

## Conclusion

In conclusion, our analysis documents the utilization of house calls by SOS doctors in the area of Attica, Greece during a 5-year period. Our experience adds to the literature the characteristics of the patients who requested the house call services as well as their health problems. There have already been several other organized systems of providing patient home visits in various countries. Among them, there are other companies and organizations of SOS doctors in Paris, Brussels, and Geneva. Despite the occasional problems that we encountered during the years of the operation of the house call services in Attica, our overall experience has been rewarding. Although our study did not compare the various types of health services and did not include an estimation of patient satisfaction, we believe that our general positive experience suggests that house calls provided by individual practitioners, physician group practices, or organized companies and organizations should re-gain their well-deserved position in the modern health care systems especially for old aged patients and patients with limited or no motility.

## Authors' contributions

GP and GT participated in the collection of the data of SOS doctors, Athens, Greece. GT is the president of the organization and takes responsibility for the integrity of the data. GP, EAK, and MEF performed the statistical analysis and wrote parts of the first version of the manuscript. All authors provided comments towards completion of the manuscript and approved its final version.

## Competing interests

EAK and MEF have no competing interests. GP and GT hold stocks in the SOS doctors, Athens, Greece.

## Pre-publication history

The pre-publication history for this paper can be accessed here:



## Figures and Tables

**Table 1:** Patient home visits by SOS doctors in Attica, Greece.

	N of house-calls*	% within category**
**Age-group **(years)		
non-recorded	2,627	2.7%
1 – 15	7,865	8.0 %
16 – 30	10,367	10.6%
31 – 45	14,735	15.0%
46 – 60	12,735	13.0 %
61 – 75	18,812	19.2%
76 – 90	26,448	27.0%
91 -	4,420	4.5%
**Patient's sex**		
Male	39,766	40.6%
Female	58,242	59.4%
**8-Hour**		
00 – 08	13,124	13.4%
08 – 16	45,542	46.5%
16 – 00	39,343	40.1%
**Month**		
January	9,764	10.0%
February	8,446	8.6%
March	10,063	10.3%
April	9,428	9.6%
May	7,946	8.1%
June	7,067	7.2%
July	6,952	7.1%
August	7,898	8.1%
September	6,353	6.5%
October	7,269	7.4%
November	7,102	7.3%
December	9,721	9.9%
**Season**		
Winter	27,931	28.5%
Spring	27,437	28.0%
Summer	21,917	22.4%
Fall	20,724	21.1%
**Year**		
2000 (Nov-Dec)	3,219	3.3%
2001	18,876	19.7%
2002	20,126	20.5%
2003	20,275	20,7%
2004	19,251	19.6%
2005 (Jan-Oct)	16,262	16.6%

*Total 98,009 house calls in the 5/year period

** All comparisons of the distributions of the studied variables had statistical significance with p < 0.001

**Table 2:** Diagnosis after evaluation of patients at home by SOS doctors in Attica, Greece.

DISEASES/DISORDERS *	**N of house-calls**	**% of all house-calls**
**Infections**	**32,975**	**33.6%**
Upper respiratory tract infections	13,375	13.7%
Gastroenteritis	5,921	6.0%
Lower respiratory tract infections	4,563	4.7%
Pharyngitis/Amygdalitis	4,389	4.5%
Urinary tract infections	1,880	1.9%
Otitis media	969	1.0%
**Cardiovascular**	**9,182**	**9.4%**
Hypertension	3,007	3.1%
Ischemic heart disease	1,979	2.0%
Hypotension/Fainting/Syncope	1,006	1.0%
Atrial fibrillation	1,000	1.0%
Other arrhythmias	1,116	1.1%
**Muscoskeletal**	**8,361**	**8.5%**
Low back pain/Sciatica	2,633	2.7%
Muscoskeletal pain	1,792	1.8%
Fractures	751	0.8%
**Gastrointestinal**	**5,557**	**5.7%**
Gastritis/Dyspepsia	1,692	1.7%
Abdominal pain	1,108	1.1%
Constipation	506	0.5%
**E.N.T.**	**3,882**	**4.0%**
Positional vertigo	2,610	2.7%
**Psychiatric**	**3,537**	**3.6%**
Anxiety disorder/Panic attack	1,859	1.9%
Depression	665	0.7%
**Neurological**	**3,162**	**3.2%**
Migraine	934	1.0%
Stroke	709	0.7%
**Genito-urinary tract**	**1,802**	**1.8%**
Renal colic	1,170	1.2%
**Respiratory tract**	**1,707**	**1.7%**
Dyspnea	600	0.6%
COPD**	582	0.6%
**Skin diseases**	**1,509**	**1.5%**
**Endocrine/Metabolic, Acid-base disturbances**	**758**	**0.8%**
**Hepatobiliary**	**566**	**0.6%**
**Allergic**	**509**	**0.5%**
**Burns-Intoxications-Bites**	**494**	**0.5%**
**Cancer**	**381**	**0.4%**
**Venous vascular**	**354**	**0.4%**
**Eye diseases**	**244**	**0.3%**
**Hematological**	**200**	**0.2%**
**Other diseases**	**1,232**	**1.3%**
**Adverse drug effects/Over-dosage**	**272**	**0.3%**
**Medical procedures**	**5,711**	**5.8%**
Placement of urinary catheters	2,789	2.9%
Placement of rhino-gastric catheters	790	0.8%
Bed-sore treatment	781	0.8%
**Required further investigation**	**6,789**	**14.3%**
**Routine physical examination**	**1,239**	**1.3%**
**Follow up examination**	**957**	**1.0%**
**No specific diagnosis recorded**	**1,420**	**1.5%**
**Missing data**	**5,209**	**5.3%**
**TOTAL**	**98,009**	**100.0%**

*In subcategories disorders presented if encountered in >500 house-calls,

**Chronic Obstructive Pulmonary Disease
